# Effectiveness of Hospital Functions for Acute Ischemic Stroke Treatment on In-Hospital Mortality: Results From a Nationwide Survey in Japan

**DOI:** 10.2188/jea.JE20140181

**Published:** 2015-08-05

**Authors:** Tetsuya Iwamoto, Hideki Hashimoto, Hiromasa Horiguchi, Hideo Yasunaga

**Affiliations:** 1 Department of Clinical Epidemiology and Health Economics, School of Public Health, The University of Tokyo, Tokyo, Japan; 1 東京大学大学院 医学系研究科 公共健康医学専攻 臨床疫学・経済学分野; 2 Department of Health and Social Behavior, School of Public Health, The University of Tokyo, Tokyo, Japan; 2 東京大学大学院 医学系研究科 公共健康医学専攻 保健社会行動学分野; 3 Department of Clinical Data Management and Research, Clinical Research Center, National Hospital Organization Headquarters, Tokyo, Japan; 3 独立行政法人国立病院機構 本部 総合研究センター 診療情報分析部

**Keywords:** ischemic stroke, acute care, stroke care unit, in-hospital mortality, instrumental variable estimation

## Abstract

**Background:**

Though evidence is limited in Japan, clinical controlled studies overseas have revealed that specialized care units are associated with better outcomes for acute stoke patients. This study aimed to examine the effectiveness of hospital functions for acute care of ischemic stroke on in-hospital mortality, with statistical accounting for referral bias.

**Methods:**

We derived data from a large Japanese claim-based inpatient database linked to the Survey of Medical Care Institutions and Hospital Report data. We compared the mortality of acute ischemic stroke patients (*n* = 41 476) in hospitals certified for acute stroke treatment with that in non-certified institutions. To adjust for potential referral bias, we used differential distance to hospitals from the patient’s residence as an instrumental variable and constructed bivariate probit models.

**Results:**

With the ordinary probit regression model, in-hospital mortality in certified hospitals was not significantly different from that in non-certified institutions. Conversely, the model with the instrumental variable method showed that admission to certified hospitals reduced in-hospital mortality by 30.7% (*P* < 0.001). This difference remained after adjusting for hospital size, volume, staffing, and intravenous use of tissue plasminogen activator.

**Conclusions:**

Comparison accounting for referral selection found that certified hospital function for acute ischemic stroke care was associated with significantly lower in-hospital mortality. Our results indicate that organized stroke care—with certified subspecialty physicians and around-the-clock availability of personnel, imaging equipment, and emergency neurosurgical procedures in an intensive stroke care unit—is effective in improving outcomes in acute ischemic stroke care.

## Abstract (ja)

**【背景】:**

脳卒中ケアユニットでの治療が急性期脳卒中患者に有効であることは海外の臨床研究により明らかにされているが、日本国内でのエビデンスは限られている。本研究は、選択バイアスの影響を考慮し、急性期脳梗塞治療体制が脳梗塞患者の入院中死亡に与える影響を検証することを目的とする。

**【方法】:**

DPCデータと医療施設調査・病院報告の個票を使用した。対象は急性期脳梗塞患者(n=41,476)とし、急性期脳卒中加算を取得した病院と取得していない病院の入院中死亡割合を比較した。選択バイアスを調整するため、患者の居住地から病院までに距離の差を操作変数としたbivariate probit モデルを使用した。

**【結果】:**

probitモデルでは、急性期脳卒中加算を取得した病院と取得していない病院の入院中死亡に有意な差が見られなかった。しかし、操作変数を用いたモデルでは、急性期脳卒中加算を取得した病院への入院が、入院中死亡率を30.7%有意に低下させていた（p<0.01）。また、病床数や患者数、医療従事者数、t-PAの使用を調整しても、同様の結果が得られた。

**【結論】:**

急性期脳卒中治療体制として急性期脳卒中加算を取得した病院への入院は、入院中死亡の低下と有意に関連がみられた。本研究結果から、脳卒中専門医の配置や24時間の人員配置、脳画像診断、脳外科的処置といった脳卒中治療体制が、脳梗塞患者のアウトカム改善に有効であることが示唆された。

## INTRODUCTION

Stroke is one of the most attributable causes of long-term disability and remains a major cause of death in developed countries, such as Japan.^1^^,^^2^ In the face of population aging and increasing numbers of stroke patients, improving acute care for stroke has become a societal need to alleviate the disease burden.

Some studies have found that hospital volume^3^^,^^4^ and adherence to treatment protocols^5^ were related to better outcomes of acute stroke care in terms of in-hospital mortality and functional recovery. More recently, based on meta-analyses of clinical trials and expert opinions, there has been consensus that organized specialty unit care for acute stroke significantly reduces mortality and morbidity.^6^^–^^8^

Based on evidence obtained overseas, the Japanese Stroke Association has issued a practice guideline. This guideline recommends that acute treatment for ischemic stroke —including administration of thrombolytic intervention using recombinant tissue plasminogen activator (rt-PA)— should be provided at institutions with experience in the appropriate subspecialties and with appropriate equipment on-hand to conduct treatment with due efficacy and safety.^9^ Following the introduction of that guideline, the Japanese Ministry of Health, Labour and Welfare introduced a new payment scheme in April 2008. Under that scheme, a bonus fee would be paid for the first day of acute-care treatment with intravenous administration of rt-PA only in hospitals that satisfied the following structural requirements: 1) certified subspecialty physicians; 2) around-the-clock availability of pharmacists and technologists in radiology and laboratory work; 3) around-the-clock availability of imaging tests, such as computed tomography and magnetic resonance imaging; 4) around-the-clock capacity for emergency neurosurgical procedures; and 5) an intensive care unit for stroke treatment.

Thus far, few studies have evaluated whether hospitals certified under the new payment scheme in Japan have produced a difference in clinical outcomes.^9^ Iihara et al conducted a questionnaire survey among board-certified training institutions for stroke care to measure the quality of organized stroke care in terms of board-certified specialty personnel, around-the-clock availability of diagnostic equipment, and specialized treatment facilities.^10^ They revealed an inverse association between the quality scores and in-hospital mortality due to stroke. However, their study sample was limited to high-performance hospitals and lacked control hospitals for comparison. Further, owing to the observational nature of the available data, there may have been selection bias through choice of institution: patients, their families, and ambulance teams may have selected large hospitals with better equipment in the hope of a better outcome. Thus, adjusting the patients’ clinical characteristics may not be sufficient to control for confounding through unobserved preferences and heterogeneous patient background. The current study attempted to overcome these limitations to estimate more precisely the clinical effectiveness of an organized acute stroke unit by means of the instrumental variable method.

## METHODS

### Data source

The primary data source for patient-level records was the Diagnosis Procedure Combination (DPC) inpatient database. The DPC is a Japanese case-mix classification system linked with a per diem inclusive payment scheme.^11^^–^^13^ As of 2010, the database included anonymous data relating to 3.19 million discharged cases from all 82 academic hospitals and 870 voluntarily participating acute-care community hospitals. Those cases amounted to approximately 45% of all inpatient admissions to acute-care beds in Japan that year. We chose data from 2010 because that was the first year, following policy implementation, for the patients’ postal codes to become available in the dataset, thereby allowing the patients’ residential location to be identified.

Details of the hospitals’ certification status and eligibility for bonus payments were obtained from the website of the Regional Bureau of Health and Welfare.^14^ We also referred to the the Survey of Medical Care Institutions and Hospital Report to obtain the location of all existing facilities in Japan as of the end of 2010 with potentially eligible equipment for acute stroke care (stroke care hospitals); that report included hospitals that did not appear in our primary data source, the DPC system.^15^ Stroke-care hospitals were identified using the following criteria: possessing care units for stroke treatment, such as internal medicine, neurology, and neurosurgery; having equipment for neurological imaging, such as computed tomography and magnetic resonance imaging; offering emergency services; and mainly offering acute care (long-term-care beds constituted <60% of all beds; psychiatric beds constituted <50% of all beds). Among 8670 institutions in Japan as of the end of 2010, 3090 (35.1%) were regarded as eligible stroke care hospitals, and 729 of the 3090 institutions were certified for bonus payments. In our sample from the DPC database, 535 were certified hospitals, and 352 of 471 non-certified hospitals met the above criteria for stroke care hospitals. We limited our sample to the 887 hospitals meeting criteria for stroke care for further analysis.

### Inclusion and exclusion criteria for stroke cases

We selected patients who were hospitalized between July 1 and December 31, 2010, for an acute attack of ischemic stroke (International Classification of Disease, 10th revision [ICD-10] codes I63$ and I693) and were hospitalized within 1 day of onset (*n* = 49 133). We excluded patients with transient ischemic events and other ambiguous conditions (eg, ICD-10 codes G45$, I675, I978). We also excluded patients who were hospitalized for over 180 days, who underwent a craniotomy, who were aged 20 or younger, and who had no functional deficit on admission (modified Rankin Scale [mRS] = 0); we did so because those patients would have had a different in-hospital trajectory from patients undergoing thrombolytic treatment. Finally, we excluded patients who obviously had a stroke attack outside their own residential area because we needed to determine in our analytical model the distance between the place of onset and the referred hospital (as described below). Patients for whom the distance between their home postal code and the postal code of the hospital was greater than the 95th percentile (33.704 km) were excluded from the data, which left 41 476 patients for further analysis (Figure).

**Figure.**
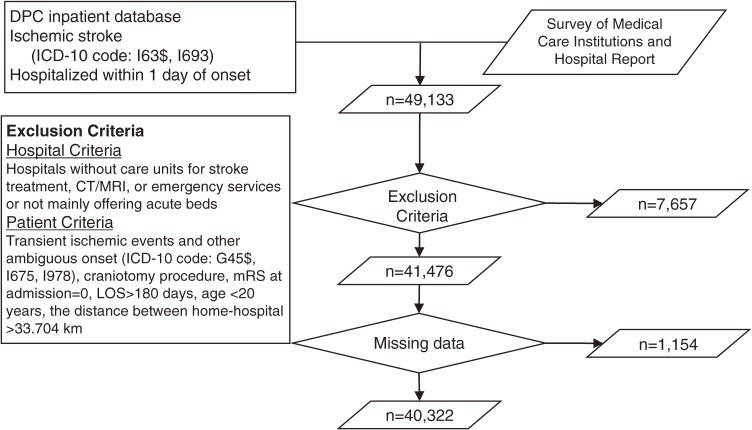
Patient selection. JCS, Japan Coma Scale; LOS, length of stay; mRS, modified Rankin Scale.

### Treatment and instrumental variables

Our treatment variable was a dichotomy as to whether or not the patient was treated in hospitals certified for bonus payment under structural requirements. However, since the transfer to a certified hospital did not occur randomly, a simple comparison would suffer seriously from referral bias. Standard risk-adjustment methods, such as multiple logistic regression models, are unsuitable for solving this type of problem because they can adjust only for measured covariates.^16^ Thus, we adopted the instrumental variable method as an alternative approach to control for unobserved heterogeneity.^17^ Following the procedure adopted in other investigations, we used the differential distance as a potentially effective instrument in the present study.^18^^–^^20^ We calculated differential distance as the difference between “road-map distance from the patient’s place of residence to the nearest certified hospital” and “road-map distance from the patient’s place of residence to the nearest stroke care hospital” to which stroke patients could have been transferred. The smaller the differential distance, the greater the chance should be of the patient being transferred to a certified hospital, and vice versa. In calculating the differential distance, we did not limit the list of certified hospitals and stroke care hospitals to those in our sample, but we used the list of all 3090 stroke-care hospitals identified in the national record noted above. We also obtained the estimated distance between the patients’ residence and the hospital to which they were actually transferred, which reflected transportation time.

Finally, we made an instrumental dummy variable for whether the differential distance was equal to or less than the median distance. Since the differential distance would be greater in a remote area with fewer hospitals, we arbitrarily defined rural and urban regions at the cut-off point of 1.5 hospitals per 100 km^2^ in a region. The median differential distance was 1.052 km in urban regions and 1.741 km in rural regions.

For these procedures, we utilized a website of Japanese address match geocoding (http://www.geocoding.jp/), which was established with Google Maps Application Program Interface and powered by Google (Mountain View, CA, USA). We identified the shortest route on the road map between a patient’s residence and a hospital using the website of a navigation system (http://plaza.umin.ac.jp/hmp/maproute.html), which was established with the Google Directions Application Program Interface and also powered by Google. Since we had only the postal codes of the patient’s residence and the hospitals, we obtained the distance as the road map distance between the centers of those postal code areas.

### Outcome variables

We used 7-day in-hospital mortality as an outcome variable to avoid the influence of different lengths of stay across hospitals. We also evaluated 14-day in-hospital mortality.

### Covariates

As covariates to predict in-hospital mortality, we included in our model age, gender, functional deficit on admission and discharge measured using the mRS,^21^ and consciousness level on admission measured with the Japan Coma Scale.^22^ The Japan Coma Scale and Glasgow Coma Scale assessments are well correlated.^23^ We also examined the intravenous administration of rt-PA within 2 days from onset, use of the thrombin inhibitor argatroban within 2 days, use of aspirin within 2 days, use of ozagrel sodium within 5 days, and use of glycerol during hospitalization; we did so because those procedures are recommended in the Japanese guideline for stroke management and have an influence on mortality.^9^

Following previous studies, we also included hospital characteristics that influence outcomes, including the number of hospital beds, the number of acute ischemic stroke patients per year, the doctor-to-patient ratio, and the nurse-to-patient ratio.^3^^–^^5^ We categorized these variables into tertiles.

### Statistical analysis

First, we used a single-equation probit model, which included certified hospital admission as a treatment variable, with patient characteristics on admission as covariates for risk adjustment. We then conducted a bivariate probit model analysis to account for selective referral using the differential distance as an instrument. The first-stage equation predicted the likelihood of certified hospital admission, with patient characteristics on admission as covariates and a dummy variable of differential distance as an instrument. The second-stage equation regressed the in-hospital mortality on certified hospital admission predicted in the first-stage equation.

If referral to certified hospitals happened at random, estimations by ordinary probit regression and that by instrumental variable method should be consistent. If referral decision was made selectively by unmeasured factors, estimations of the two models should be different, the significance of which can be tested by the Hausman test. Thus, we presented both results of a single-equation probit model and a bivariate probit model using an instrumental variable.

To better identify factors influencing the outcome, we conducted additional bivariate probit models with a reduced or added set of covariates. We excluded patients who were treated by intravenous administration of rt-PA and repeated the analysis to test whether rt-PA was an influential care process on the observed difference between certified and non-certified hospitals. We also included in the model structural characteristics of hospitals (such as hospital volume, physician-to-patient ratio, and nurse-to-patient ratio) and treatment processes mentioned earlier in the second-stage equation to test whether these structural and process factors explained the difference between certified and non-certified hospitals.

We further examined the robustness of the result with several approaches. We excluded patients whose differential distance to the second-nearest stroke care hospital was twice that to the nearest hospital. In such a case, the patient had virtually no choice but to be transferred to the nearest hospital. Harris and Remler^24^ noted that including the “no choice case” would bias the estimation of the treatment effect. We used a chi-square test to compare the proportions and a *t* test to compare the average values between the groups.

Since the bivariate probit model does not provide suitable statistical tests for relevance and strength of the instrument, we additionally performed two-stage least-squares regression analysis, which was regressed on the same set of covariates.^25^^,^^26^ We used a robust standard error following White’s method.^27^ We conducted the Hausman test and *F* test to examine the relevance and strength of the instrument, respectively.^28^ A *P* value <0.05 was considered significant. We performed all analyses using Stata 11.0 (StataCorp, College Station, TX, USA).

### Ethical considerations

Study approval was obtained from the institutional review board at the University of Tokyo. Owing to the anonymous nature of the secondary data analysis, the need for informed consent was waived.

## RESULTS

### Patient characteristics

In all, 29 310 patients were transferred to certified hospitals, and 11 012 patients were transferred to non-certified acute-care hospitals. Patients who were transferred to certified hospitals were more frequently younger, male, and more likely to have impaired consciousness on admission (Table 1). The chi-square test showed that 7-day in-hospital mortality was significantly different between the two hospital categories (2.7% vs 2.3%, *P* = 0.014).

**Table 1.** Patient characteristics by transfer destination

	Certified hospitals	Non-certified hospitals	*P* value
Number of patients	29 310	11 012	
Age (mean [SD]), years	74.1 (12.0)	75.3 (12.0)	<0.001
Male sex (%)	58.3%	55.2%	<0.001
Functional deficit on admission (%)			
mRS = 1	11.3%	12.6%	<0.001
mRS = 2–3, JCS = 0–3	34.8%	33.2%	
mRS = 4–5, JCS = 0–3	39.0%	40.3%	
mRS = 4–5, JCS = 10–30	9.6%	8.9%	
mRS = 4–5, JCS = 100–300	5.3%	5.0%	
LOS (mean [SD]), days	25.6 (23.8)	28.5 (29.3)	<0.001
7-day in-hospital mortality (%)	2.7%	2.3%	0.017
Hospital size^a^ (%)			
Low	12.7%	69.7%	<0.001
Medium	39.2%	20.9%	
High	48.1%	9.3%	
Hospital volume^b^ (%)			
Low	25.8%	52.5%	<0.001
Medium	36.6%	21.4%	
High	37.6%	26.1%	
Physician-to-patient ratio^c^ (%)			
Low	20.9%	65.1%	<0.001
Medium	45.2%	24.4%	
High	33.9%	10.6%	
Nurse-to-patient ratio^d^ (%)			
Low	27.7%	53.5%	<0.001
Medium	36.8%	21.8%	
High	35.4%	24.7%	

JCS, Japan Coma Scale; LOS, length of stay; mRS, modified Rankin Scale; SD, standard deviation.

^a^Hospital size: low, <300 beds; medium, 300–500 beds; high, ≥500 beds.

^b^Hospital volume: low, <120 patients per year; medium, 120–199 patients per year; high, ≥200 patients per year.

^c^Physician-to-patient ratio: low, <20 physicians per 100 patients; medium, 20–29.9 physicians per 100 patients; high, ≥30 physicians per 100 patients.

^d^Nurse-to-patient ratio: low, <90 nurses per 100 patients; medium, 90–104.9 nurses per 100 patients; high, ≥105 nurses per 100 patients.

Table 2 shows the patients’ characteristics compared using the instrument variable of differential distance. There was no significant difference between the two groups with respect to age, gender, or functional levels on admission. However, the proportion of patients transferred to a certified hospital was significantly different (84.9% vs 60.5%, *P* < 0.001), which suggests that differential distance was valid as an instrumental variable. Patients whose differential distance was equal to or less than the median (or who should more likely have been admitted to a certified hospital) had a lower mortality rate than those with a greater differential distance (2.4% vs 2.8%, *P* < 0.001).

**Table 2.** Patient characteristics by differential distance

	Smaller differential distance^a^	Greater differential distance^b^	*P* value
Number of patients	20 142	20 180	
Age (mean [SD]), years	74.5 (12.1)	74.4 (12.0)	0.273
Male sex (%)	57.5%	57.5%	0.991
Functional deficit on admission (%)			
mRS = 1	11.7%	11.6%	0.238
mRS = 2–3, JCS = 0–3	34.8%	33.9%	
mRS = 4–5, JCS = 0–3	39.2%	39.6%	
mRS = 4–5, JCS = 10–30	9.2%	9.5%	
mRS = 4–5, JCS = 100–300	5.1%	5.4%	
Certified hospitals (%)	84.9%	60.5%	<0.001
LOS (mean [SD]), days	26.2 (25.2)	26.6 (25.7)	0.119
7-day in-hospital mortality (%)	2.4%	2.8%	0.005
Hospital size^c^ (%)			
Low	21.4%	35.1%	<0.001
Medium	37.9%	30.6%	
High	40.7%	34.4%	
Hospital volume^d^ (%)			
Low	31.6%	34.7%	<0.001
Medium	35.0%	29.9%	
High	33.5%	35.5%	
Physician-to-patient ratio^e^ (%)			
Low	28.6%	37.2%	<0.001
Medium	43.2%	35.8%	
High	28.2%	26.9%	
Nurse-to-patient ratio^f^ (%)			
Low	31.6%	37.9%	<0.001
Medium	35.7%	29.7%	
High	32.7%	32.3%	

JCS, Japan Coma Scale; LOS, length of stay; mRS, modified Rankin Scale; SD, standard deviation.

^a^Smaller differential distance: differential distance of ≤1.052 km in urban regions and ≤1.741 km in rural regions.

^b^Greater differential distance: differential distance of >1.052 km in urban regions and >1.741 km in rural regions.

^c^Hospital size: low, <300 beds; medium, 300–500 beds; high, ≥500 beds.

^d^Hospital volume: low, <120 patients per year; medium, 120–199 patients per year; high, ≥200 patients per year.

^e^Physician-to-patient ratio: low, <20 physicians per 100 patients; medium, 20–29.9 physicians per 100 patients; high, ≥30 physicians per 100 patients.

^f^Nurse-to-patient ratio: low, <90 nurses per 100 patients; medium, 90–104.9 nurses per 100 patients; high, ≥105 nurses per 100 patients.

### Effect of certified hospitals on in-hospital mortality

The results of the single probit and bivariate probit analysis appear in Table 3. The single probit analysis showed that older age and functional severity were significantly and positively associated with in-hospital mortality. With this model, transfer to a certified hospital was positively, but not significantly, associated with mortality. Conversely, the bivariate probit model showed that transfer to a certified hospital was significantly and negatively associated with 7-day in-hospital mortality (coefficient = −0.370, *P* < 0.001), which suggests that transfer to a certified hospital reduced mortality by 37.0%.

**Table 3.** Results of single probit and bivariate probit model on 7-day in-hospital mortality

	Single probit model		Bivariate probit model	
	
	Coefficient	Standard error	Coefficient	Standard error
Certified stroke hospital	0.078	0.035	−0.370***	0.139
Sex (male = 1)	−0.061*	0.032	−0.055*	0.084
Age	0.003**	0.001	0.003*	0.001
Functional deficit on admission				
mRS = 1				
mRS = 2–3, JCS = 0–3	0.298**	0.143	0.302**	0.140
mRS = 4–5, JCS = 0–3	0.929***	0.136	0.922***	0.133
mRS = 4–5, JCS = 10–30	1.562***	0.137	1.555***	0.135
mRS = 4–5, JCS = 100–300	2.223***	0.138	2.206***	0.138
First-stage regression				
Smaller differential distance^a^	—	—	0.769***	0.014

JCS, Japan Coma Scale; mRS, modified Rankin Scale.

^a^Smaller differential distance, differential distance of ≤1.052 km in urban regions and ≤1.741 km in rural regions.

*P < 0.1, **P < 0.05, ***P < 0.01.

The results of the additional bivariate probit model with the added set of covariates are shown in Table 4. The coefficient was −0.429 (*P* < 0.01) when all hospital structural factors were added to the model. We repeated the analysis using 14-day mortality as the outcome but obtained the same results (Table 4). When treatment processes were added to the model, the coefficient showed a similar value (Table 4, model 3). After excluding patients treated with intravenous administration of rt-PA, the coefficient of certified hospital treatment remained significantly negative (data not shown). After excluding patients whose residences were far from the second-nearest hospital relative to the nearest hospital (the “no choice cases”), the coefficient of the certified hospital retained the same point estimate, although the confidence interval was greater owing to the smaller sample size (data not shown).

**Table 4.** Effect of certified hospital admission on mortality by instrument variable method, adjusting for hospital characteristics and treatment processes

	Model 1^a^		Model 2^b^		Model 3^c^	
		
	Coefficient	95% CI	Coefficient	95% CI	Coefficient	95% CI
7-day in-hospital mortality	−0.370***	[−0.643, −0.097]	−0.429***	[−0.710, −0.148]	−0.430***	[−0.723, −0.136]
14-day in-hospital mortality	−0.266**	[−0.500, −0.033]	−0.279***	[−0.517, −0.040]	−0.259**	[−0.505, −0.013]

CI, confidence interval.

^a^Model 1, adjusted for age, sex, and functional deficit on admission.

^b^Model 2, adjusted for age, sex, functional deficit on admission, and all hospital structural factors (hospital size, hospital volume, doctor-to-patients ratio, nurse-to-patients ratio).

^c^Model 3, adjusted for age, sex, functional deficit on admission, and use of treatment processes (thrombin inhibitor argatroban within 2 days, aspirin within 2 days, ozagrel sodium within 5 days, and glycerol during the hospitalization).

*P < 0.1, **P < 0.05, ***P < 0.01.

### Sensitivity analyses

The results of the two-stage least-squares regression analysis estimates similarly showed a significant and negative coefficient of certification status (data not shown). The Hausman test verified the endogeneity of the hospitals’ certification status (*P* = 0.002), and it supported the use of the instrumental variable estimation. The F statistics from the first-stage regression indicated that the instrumental variable had sufficient strength for predicting admission to certified hospitals (F [1, 40 314] = 3290, *P* < 0.001). These results suggest that differential distance was valid as an instrumental variable.

## DISCUSSION

Our results reveal that acute ischemic stroke patients who were transferred to institutions certified for organized stroke care exhibited significantly lower in-hospital mortality than those treated in non-certified institutions. The differences between a single-equation probit estimate and a bivariate probit estimate was significant in the Hausman test, suggesting that referral to certified hospitals was made selectively by unmeasured factors, and the results obtained via the instrumental variable method should be adopted as a less biased estimation of the effect. Certified hospitals were also significantly larger, had a greater case volume, and were staffed with more physicians and nurses. When we further adjusted for hospital volume, hospital size, staffing of physicians and nurses per bed, and use of treatment processes recommended in the clinical guideline, the superior outcome in certified hospitals remained significant. Furthermore, patients in certified institutions were more likely to receive intravenous administration of rt-PA; however, the lower in-hospital mortality in certified hospitals persisted even after we limited the analysis to cases without that therapy. The results strongly suggest that the superior outcomes in certified institutions were independent of rt-PA administration, hospital size, and staffing. Recent measures to improve the quality of acute care for stroke have made major efforts in terms of infrastructure and personnel allocation, so as to secure around-the-clock readiness for treatment in comprehensive stroke care centers.^6^^,^^7^^,^^10^ Our present findings therefore indicate that the excellence of certified hospitals derives from the around-the-clock management of acute emergency stroke.

Our results provide an empirical rationale for the bonus payments to certified institutions towards achieving better outcomes. Although the current payment scheme provides reimbursement only for cases with intravenous administration of rt-PA on the first day, our results indicated that the benefits of certification should be extended to all acute stroke patients treated in certified institutions. Since the around-the-clock allocation of personnel and equipment demands resource investment, financial support should be extended to properly reward hospitals that implement quality improvement in acute stroke care.

The strengths of this study include the large dataset of stroke patients and the use of the instrumental variable method to account for referral bias. However, our study has several limitations, which require careful consideration. First, the DPC database included limited information on stroke severity, and our risk adjustment may have been insufficient. However, since the instrument variable successfully balanced the observed patient characteristics between the hospital groups, we believe that the instrument would work similarly on unmeasured patient characteristics. Second, we chose not to analyze functional outcome on discharge, despite it being an important outcome measure of treatment effectiveness in acute stroke. The DPC database did include functional deficit at the time of discharge as measured with the mRS. However, a simple comparison of functional status on discharge is not relevant because of differences in the average length of stay and referral patterns after discharge between the hospital groups. Addressing the question of whether treatment in certified hospitals leads to better functional outcomes would require data collection extended to the long-term-care stage, and that is a matter for future research. Third, we focused on ischemic stroke in this study, although the related hospital functions may have a different impact on other types of stroke.^10^ Fourth, we did not include variables related to rehabilitation for acute stroke. In our dataset, the likelihood of very early rehabilitation was not significantly different between certified and non-certified hospitals. Finally, participation in the DPC system is voluntarily, and hospitals with acute tertiary care were more likely to have participated. In Japan, the majority of stroke patients are treated in smaller, less equipped hospitals. Thus, our results may not be generalizable to those small hospitals. Extended data collection to smaller hospitals will be necessary to reveal the treatment quality for acute stroke in such smaller institutions.

### Conclusions

We found that transfer to certified institutions for acute ischemic stroke treatment was significantly associated with lower in-hospital mortality. Our results indicate that organized stroke care—with certified subspecialty physicians and around-the-clock availability of personnel, imaging equipment, and access to emergency neurosurgical procedures in an intensive stroke care unit—is effective in improving outcomes in acute ischemic stroke care.

## ONLINE ONLY MATERIAL

Abstract in Japanese.
