# Supplementary material for: Effectiveness of Hospital Functions for Acute Ischemic Stroke Treatment on In-Hospital Mortality: Results From a Nationwide Survey in Japan
Source: J Epidemiol. 2015 Aug 5;25(8):522–8. doi: 10.2188/jea.JE20140181 (PMC4517990; doi:10.2188/jea.JE20140181)
Supplement: Abstract in Japanese. [file je-25-522-s001.pdf]

急性期脳梗塞治療体制が脳梗塞患者の入院中死亡に与える影響

岩本哲哉[1,2]、橋本英樹[2]、堀口裕正[3]、康永秀生[1]

[1] 東京大学大学院　医学系研究科　公共健康医学専攻　臨床疫学・経済学分野

[2] 東京大学大学院　医学系研究科　公共健康医学専攻　保健社会行動学分野

[3] 独立行政法人国立病院機構　本部　総合研究センター　診療情報分析部

【背景】脳卒中ケアユニットでの治療が急性期脳卒中患者に有効であることは海外の臨床研究により明らかにされているが、日本国内でのエビデンスは限られている。本研究は、選択バイアスの影響を考慮し、急性期脳梗塞治療体制が脳梗塞患者の入院中死亡に与える影響を検証することを目的とする。

【方法】DPC データと医療施設調査・病院報告の個票を使用した。対象は急性期脳梗塞患者(n=41,476)とし、急性期脳卒中加算を取得した病院と取得していない病院の入院中死亡割合を比較した。選択バイアスを調整するため、患者の居住地から病院までに距離の差を操作変数とした bivariate probit モデルを使用した。

【結果】probit モデルでは、急性期脳卒中加算を取得した病院と取得していない病院の入院中死亡に有意な差が見られなかった。しかし、操作変数を用いたモデルでは、急性期脳卒中加算を取得した病院への入院が、入院中死亡率を 30.7%有意に低下させていた（$p<0.01$）。また、病床数や患者数、医療従事者数、t-PA の使用を調整しても、同様の結果が得られた。

【結論】急性期脳卒中治療体制として急性期脳卒中加算を取得した病院への入院は、入院中死亡の低下と有意に関連がみられた。本研究結果から、脳卒中専門医の配置や 24 時間の人員配置、脳画像診断、脳外科的処置といった脳卒中治療体制が、脳梗塞患者のアウトカム改善に有効であることが示唆された。

キーワード: 脳梗塞、急性期治療、脳卒中ケアユニット、入院中死亡、操作変数法
